# Prostaglandins in biofluids in pregnancy and labour: A systematic review

**DOI:** 10.1371/journal.pone.0260115

**Published:** 2021-11-18

**Authors:** Eilidh M. Wood, Kylie K. Hornaday, Donna M. Slater

**Affiliations:** 1 Department of Physiology and Pharmacology, Cumming School of Medicine, University of Calgary, Calgary, Alberta, Canada; 2 Department of Obstetrics and Gynecology, Cumming School of Medicine, University of Calgary, Calgary, Alberta, Canada; John Hunter Hospital, AUSTRALIA

## Abstract

Prostaglandins are thought to be important mediators in the initiation of human labour, however the evidence supporting this is not entirely clear. Determining how, and which, prostaglandins change during pregnancy and labour may provide insight into mechanisms governing labour initiation and the potential to predict timing of labour onset. The current study systematically searched the existing scientific literature to determine how biofluid levels of prostaglandins change throughout pregnancy before and during labour, and whether prostaglandins and/or their metabolites may be useful for prediction of labour. The databases EMBASE and MEDLINE were searched for English-language articles on prostaglandins measured in plasma, serum, amniotic fluid, or urine during pregnancy and/or spontaneous labour. Studies were assessed for quality and risk of bias and a qualitative summary of included studies was generated. Our review identified 83 studies published between 1968–2021 that met the inclusion criteria. As measured in amniotic fluid, levels of PGE_2_, along with PGF_2α_ and its metabolite 13,14-dihydro-15-keto-PGF_2α_ were reported higher in labour compared to non-labour. In blood, only 13,14-dihydro-15-keto-PGF_2α_ was reported higher in labour. Additionally, PGF_2α_, PGF_1α_, and PGE_2_ were reported to increase in amniotic fluid as pregnancy progressed, though this pattern was not consistent in plasma. Overall, the evidence supporting changes in prostaglandin levels in these biofluids remains unclear. An important limitation is the lack of data on the complexity of the prostaglandin pathway outside of the PGE and PGF families. Future studies using new methodologies capable of co-assessing multiple prostaglandins and metabolites, in large, well-defined populations, will help provide more insight as to the identification of exactly which prostaglandins and/or metabolites consistently change with labour. Revisiting and revising our understanding of the prostaglandins may provide better targets for clinical monitoring of pregnancies. This study was supported by the Canadian Institutes of Health Research.

## Introduction

It is widely believed that prostaglandins are important in the initiation of human labour [1]. Multiple studies have documented increased expression of cyclooxygenases, key enzymes in prostaglandin synthesis, in gestational tissues with the onset of labour, however, this has not been consistently observed [2]. Additionally, prostaglandins are present in maternal blood, urine, and amniotic fluid during pregnancy [3], however, the evidence supporting or refuting their role in labour is conflicting. Prostaglandins are known to affect uterine contractility and cervical ripening [4] and have thus been successfully used for labour induction since the late 1960’s, though the use of prostaglandin synthesis inhibitors for prevention of preterm birth has been minimally successful and is associated with various fetal side effects [5]. Since their discovery in the 1930s, prostaglandins and their synthesis and metabolism are now known to be highly complex, which may contribute to these inconsistent outcomes seen during clinical targeting of this pathway. Aside from providing insight into labour processes, the presence of prostaglandins in peripheral tissues offers the potential for minimally invasive early prediction of labour onset and the ability to distinguish between true and false labour, which remains an ongoing clinical challenge [6]. Additionally, it has been suggested that biomarkers predictive of term labour (>37 weeks gestation) may also be useful for prediction of preterm labour (<37 weeks gestation), as both processes share common physiological changes involving cervical ripening, uterine contractions, and membrane rupture [7]. In 2010, preterm birth was estimated to occur in approximately 11% of all pregnancies and remains the leading cause of neonatal mortality worldwide [8], yet there is a lack of objective measures available to assess risk of premature delivery. Accurate prediction of term and preterm labour would allow for more informed patient planning and more efficient use of healthcare resources, for example, by reducing unnecessary hospitalizations and interventions. Despite evidence to suggest a role for prostaglandins in pregnancy and labour, literature defining the complexities of the pathway remain inconclusive and inconsistent. Therefore, we have systematically reviewed the scientific literature with the aim of answering three main questions to find evidence that either supports or refutes a role for prostaglandins in the initiation of labour: 1) Are prostaglandins or their metabolites detectable in biofluids in higher amounts in labour vs not in labour? 2) Are prostaglandins or their metabolites detected in increasing amounts prior to the onset of labour? And 3) Are prostaglandins or their metabolites present in urine, blood, or amniotic fluid predictive of preterm labour?

## Methods

This systematic review was conducted and reported following the recommendations of the Preferred Reporting Items for Systematic Review and Meta-Analyses (PRISMA). The protocol is available upon request. This review was not registered.

### Information sources

The databases MEDLINE and EMBASE were searched for records. Additionally, the reference lists of eligible studies and relevant review articles were manually searched.

### Search strategy

The search strategy included the key words “prostaglandins” AND “obstetric labor” AND (“amniotic fluid” OR “blood” OR “urine”) as well as synonyms, related alternatives, and Medical Subject Heading (MeSH) terms as relevant. The searches were limited to human studies. Full details of the search terms for each database are given in S1 and S2 Tables. Citations retrieved from the initial search were downloaded into a reference manager (EndNote X9) and duplicates were removed. Two reviewers (EW and SLW) independently reviewed abstracts and removed those not relevant to the research questions. Following retrieval of full-text articles, both reviewers assessed the remaining citations against the eligibility criteria. Studies excluded at this level were sorted based on reason for exclusion. Disagreements were resolved by discussion until consensus was reached.

### Inclusion/Exclusion criteria

Primary study journal articles examining endogenous prostaglandins in blood, amniotic fluid, and/or urine during pregnancy and spontaneous labour were included in this review. Studies were excluded if the study was on animals, the study was examining exogenous prostaglandins for induction of labour or if participants experienced spontaneous abortion (prior to 20 weeks). As well, studies which only had samples collected following delivery were excluded. Publications with incomplete information (i.e., conference abstracts) were excluded. Only studies written in English or with an available English translation were included. The search did not include a time restriction, however, the databases MEDLINE and EMBASE include literature published since 1946 and 1947 respectively. The search was initially conducted on May 19, 2020 and was repeated on August 20, 2021.

### Selection process/Data extraction

The following information was extracted by one reviewer (EW) from each of the final selected studies: population examined, sample number, type of biofluid collected, method of testing and measurement, metabolites/prostaglandins measured, time of sample collection, country of study origin, available measures of central tendency and variance, and major findings of the study.

### Quality assessment

Studies were assessed for quality and risk of bias using a quality assessment tool (Table 1) adapted from Hadley et al. [9] for assessment of basic science research. Full details of the rubric can be found in Table 1. Studies were scored between 0–9. All studies were scored independently by two investigators (EW and KH) and disagreements in scores were resolved by discussion.

**Table 1:** Quality assessment rubric.

Quality assessment	1 point	0 points	N/A
1) Question/objective sufficiently described?	Primary study question or objective is clearly stated	Unclear question/objective or no question/objective	
2) Design appropriate to answer study question?	Study design is clearly stated and makes sense according to the study question/objective	E.g. uses convenient samples or study does not give enough information to determine study design	
3) Methods described in sufficient detail to allow for study to be replicated?	Samples, reagents, assay used to measure prostaglandins are sufficiently described, methods for sample collection are clearly described	Some information missing or no information/ insufficient information is given on samples, reagents, assays, methods for sample collection	
4) Researchers used blinding?	Yes	No	
5) Sample number sufficient for internal validity?	Study has pre-planned sample size and/or power analysis or confidence intervals suggest sufficient sample size	No power analysis or confidence intervals suggest insufficient sample size	
6) Appropriate negative controls?	Control group is appropriate to answer study question	Controls are from a clearly different population	
7) Appropriate statistical analysis?	There is a comparison of means with appropriate transformations of data	No statistical analysis provided	
8) Results reported in sufficient detail?	Results match methods i.e. all prostaglandins measured are reported on	Some measurements missing from results	
9) Do the results support the conclusion?	Conclusion makes sense given results and answers primary study question/objective	Conclusion is overstated based on results or not related to main study question and main results	

### Data synthesis

A qualitative summary was generated, and tables were created with the main results from each study. No pooled analysis was performed.

## Results

### Studies identified

**Fig 1:**
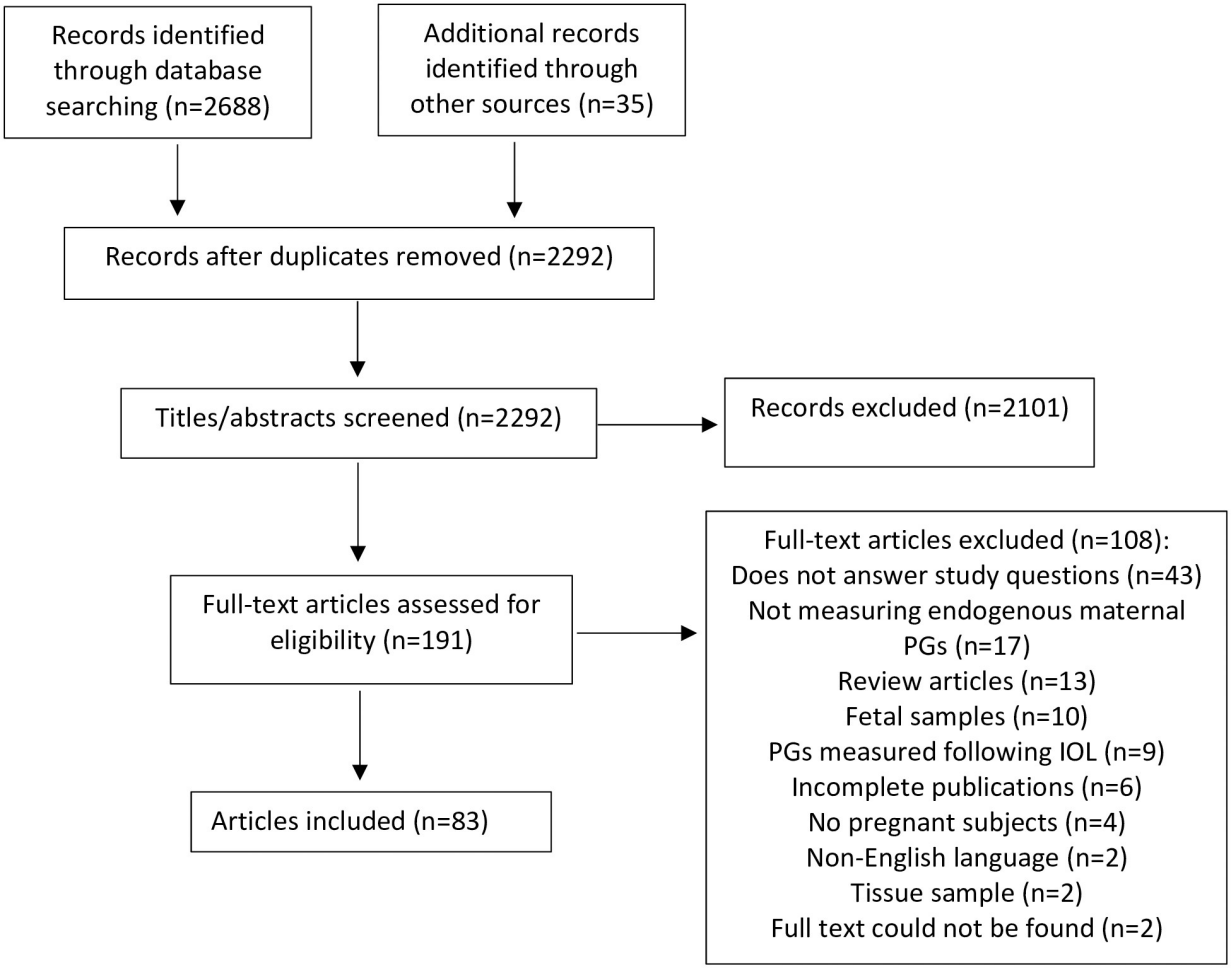
PRISMA diagram. Abbreviations: PG = prostaglandin, IOL = induction of labour.

The electronic search returned 2257 unique records after removal of duplicates from 2688 records. 2101 records were removed at the title/abstract level, leaving 156 records for assessment at the full-text level. Hand search of reference lists yielded an additional 35 records for review, resulting in a total of 191 full text records. Of the records assessed at the full text level, 108 were excluded, leaving n = 83 studies for inclusion in this review (Fig 1).

### Main characteristics of studies

**Fig 2:**
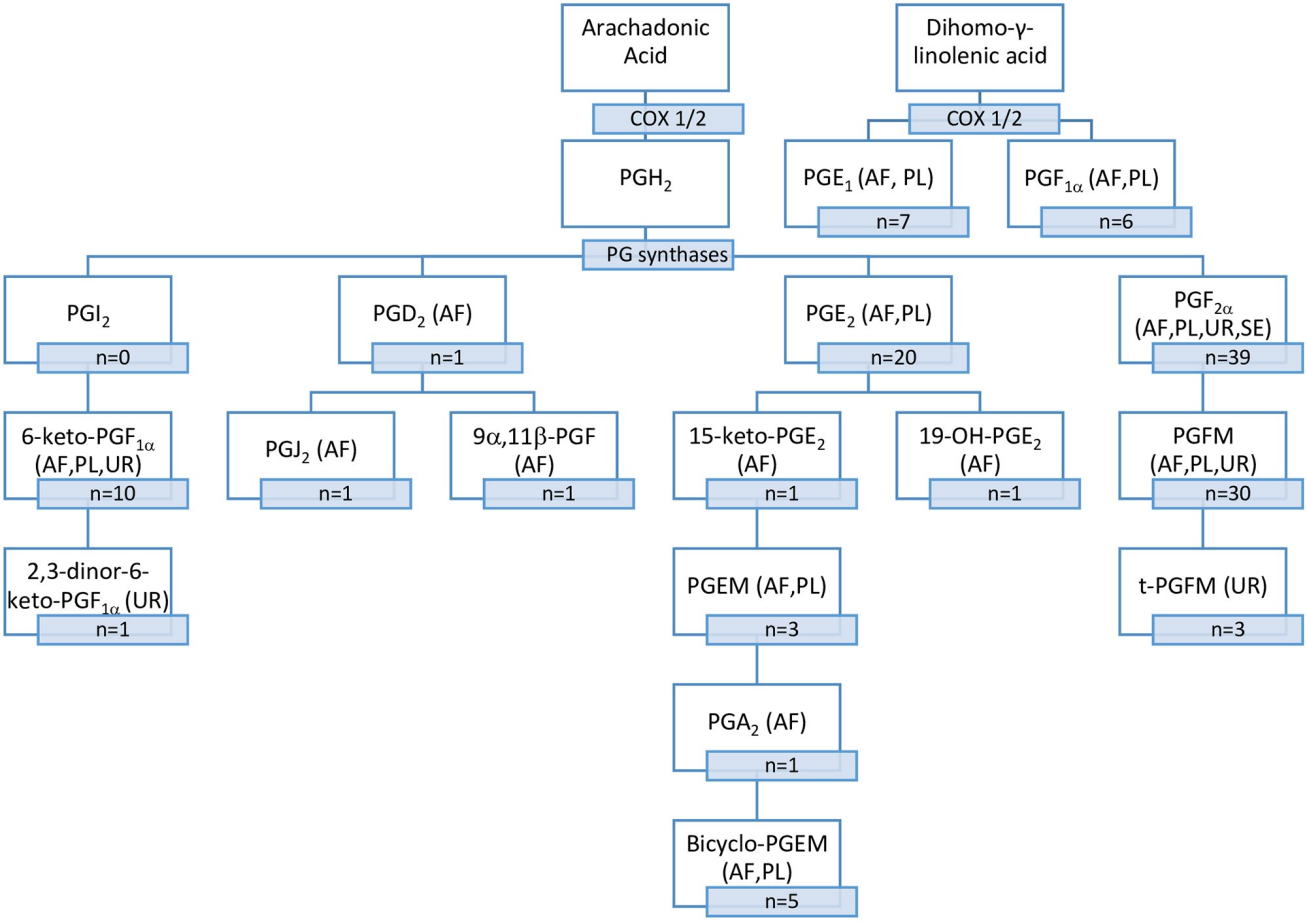
Prostaglandin metabolism pathway. n denotes the number of studies that measured the prostaglandin/metabolite. Abbreviations: AF = amniotic fluid, PL = plasma, UR = urine, SE = serum, COX 1/2 = cyclooxygenase 1/2, PGFM = 13,14-dihydro-15-keto-PGF_2α_, PGEM = 13,14-dihydro-15-keto-PGE_2_, bicyclo-PGEM = 11-deoxy-13,14-dihydro-15-keto-11,16-bicyclo PGE_2_, t-PGFM = 5α,7α-dihydroxy-11-keto tetranor-prostane-1,16-dioic acid.

Summaries of the main characteristics and relevant findings of the included studies can be found in Table 2 (presented in chronological order). Of the 83 studies, most assessed only one biofluid, (34 plasma, 32 amniotic fluid, 8 urine, 4 serum) while 6 studies assessed multiple biofluids. The range of prostaglandins and metabolites investigated included PGF_2α_, PGF_1α_, 13,14-dihydro-15-keto-PGF_2α_ (PGFM), 5α,7α-dihydroxy-11-keto-tetranor-prostane-1,16-dioic acid (t-PGFM), PGE_1_, PGE_2_, 15-keto-PGE_2_, 13,14-dihydro-15-keto-PGE_2_ (PGEM), 11-deoxy-13,14-dihydro-15-keto-11,16-bicyclo-PGE_2_ (bicyclo-PGEM), 6-keto-PGF_1α_, 2,3-dinor-6-keto-PGF_1α_, PGA_2_, PGD_2_, PGJ_2_, 19-OH-PGE_2_, and 9α,11β-PGF_2_. Prostaglandins and the corresponding metabolites measured are described in Fig 2. In addition, many older studies used measurement techniques which were unable to differentiate between subcategories of prostaglandins and therefore reported levels of PGE or PGF. The range of prostaglandin concentrations reported using different measurement techniques are shown in Table 3.

**Table 2:** Main characteristics of studies.

Study	Method	Sample Size	Biofluid	PG/Metabolite	Relevant Findings
Karim 1968 [10]	TLC and biological assay	n = 42 NL, n = 10 TL	plasma	PGF_2α_, PGF_1α_, PGE_1_, PGE_2_	NL < LOD
				PGF_2α_	higher at delivery than 1st stage labour
Brummer 1972 [11]	RIA	n = 40 NL, n = 46 L	serum	PGF_2α_	L>TNL
					late pregnancy similar to nonpregnant
					increased through 1st stage labour (early-late), then decreased in 2nd stage
Gutierrez-Cernosek & Levine 1972 [12]	RIA	n = 10 1st TM	serum	PGF_2α_	peaked at 2nd TM, decreased to nonpregnant levels at term
		n = 52 2nd TM			
		n = 54 3rd TM			
		n = 9 serial (14-40wks)			
Brummer 1973 [13]	unknown	n = 13 1st TM	serum	PGF_2α_	decreased in 2nd TM, plateaued in 3rd TM
		n = 40 2nd TM			
		n = 75 3rd TM			
Brummer & Craft 1973 [14]	RIA	n = 58 L	serum	PGF_2α_	highest in 1st stage labour, decreased in 2nd stage and remained low
		n = 7 serial L			
Hertelendy et al 1973 [15]	RIA	n = 8 PTNL	plasma	PGE	<32wks pregnant similar to nonpregnant
		n = 32 L			increased through 1st stage labour (early-late), then decreased in 2nd stage
Keirse & Turnbull 1973 [16]	GC	n = 12 TNL, n = 38 TL	AF	PGE_2_	TL>TNL
					increased through 1st stage labour
				PGE_1_	<LOD
Salmon & Army 1973 [17]	RIA	n = 57	AF	PGF_2α_	L>TNL
					constant through 2nd and 3rd TM, rise after 36wks
					spike during 1st stage labour
Challis et al 1974 [18]	RIA	n = 4 TNL, n = 9 TL	plasma	PGF	TL>TNL (nonsignificant)
Green et al 1974 [19]	GC-MS	n = 2 TL	plasma	PGF_2α_	no correlation with stage of labour
		n = 5 term serial		PGFM	TL>TNL
					increased through 1st stage labour
Hamberg 1974 [20]	RIA?	n = 3 serial (9-40wks)	urine	t-PGFM	increased with GA, peaked at term
		n = 8 TNL, n = 1TL			TL>TNL
Hennam et al 1974 [21]	RIA	n = 13 1st TM	plasma	PGF_2α_	levels lowest at 2nd TM compared to 1st and 3rd
		n = 10 2nd TM			L>3rd TM
		n = 20 3rd TM			
		n = 99 L			
Hibbard et al 1974 [22]	RIA	n = 42 TNL, n = 13 TL	AF	PGF_2α_	TL>TNL
					increased with GA after 36 weeks
		n = 22 PTNL			64% <LOD
Hillier et al 1974 [23]	RIA	n = 11 TNL, n = 5 TL	AF	PGF_2α_	TL>TNL
					increased with labour stage
			plasma		no correlation with labour stage
Keirse et al 1974 [24]	RIA and GC	n = 20 TNL, n = 26 TL	AF	PGF	TL>TNL
		n = 8 PTNL			TNL>PTNL
					increased through 1st stage labour
MacDonald et al 1974 [25]	RIA	n = 6 NL, n = 6 L	AF	PGF_2α_	L>NL
Singh & Zuspan 1974 [26]	PC and TD	n = 6	AF	PGF_2α_, PGF_1α_, PGE_1_, PGE_2_	constant from 24-36wks, increase in labour
Hillier et al 1975 [27]	RIA	n = 13 TNL, n =? TL	AF	PGF	increased with labour stage, peaked before delivery
		n = 8 PTNL			increased from 2nd TM to term
Johnson et al 1975 [28]	RIA	n = 38 NL, n = 8 L	AF	PGF_2α_	L>NL
		n = 11 PTNL			3rd TM > 2nd TM
		n = 33 NL, n = 99 L	plasma		no difference between NL and L
		n = 15 PTNL			no pattern with labour
Pokoly & Jordan 1975 [29]	RIA	n = 6 TNL, n = 2 TL (CS)	AF	PGF	TL>TNL for CS only
		n = 4 TNL, n = 13 TL	plasma		no difference between NL and L
			AF	PGE	TL>TNL (nonsignificant)
			plasma		no difference between NL and L
Dray & Frydman 1976 [30]	RIA	n = 24 NL, n = 37 L	AF	PGF_2α_	L>NL
		n = 19 PTNL			higher in late 3rd TM than early 3rd TM
					increased with labour stage
				PGE_2_	L>TNL
					<LOD before 24wks, increased to 36wks, then remained constant to term
					increased with labour stage
				PGE_1_	<LOD
Granstrom & Kindahl 1976 [31]	RIA	n = 1 term serial	urine	t-PGFM	TL>TNL
					late 3rd TM > nonpregnant
Keirse et al 1977 [32]	RIA	n = 40 TNL, n = 46 TL	AF	PGF, PGFM	TL>TNL
					increased through 1st stage labour
Kinoshita et al 1977 [33]	RIA	n = 7 TNL, n = 10 TL	AF	PGF_2α_	TL>TNL
				PGE_1_	no difference between TNL and TL
		n = 10 TL, n = 10 TNL	plasma	PGF_2α_	no difference between TNL and TL
		n = 10 3rd TM serial			no pattern with gestation in 3rd TM
				PGE_1_	no difference between TNL and TL
					no pattern with gestation in 3rd TM
					no correlation with labour stage
TambyRaja et al 1977 [34]	RIA	n = 27 PTL	AF	PGF_2α_	increased through 1st stage labour
Haning et al 1978 [35]	RIA	n = 4 TNL, n = 8 TL	plasma	PGFM	TL>TNL
Mitchell et al 1978 [36]	RIA	n = 13 NL, n = 10 L	plasma	PGF, PGE	L > NL
		n = 7 PTL			no correlation with stage of labour
				PGFM	L > NL
					no difference with PTL and NL
					increased with labour stage
Nieder & Augustin 1978 [37]	RIA	n = 34	AF	PGF_2α_, PGE	increased from 31wks to term, steeper after 36wks
			plasma		no correlation with GA
Zuckerman et al 1978 [38]	RIA	n = 5 L	plasma	PGF_2α_	lower in 1st stage labour than 2nd or 3rd
					peaked at delivery and at placental separation
Ghodaonkar et al 1979 [39]	RIA	n = 2 serial (20-40wks)	plasma	PGFM	no pattern with gestation
		n = 14 TL			increased in 2nd and 3rd stages of labour
Mitchell et al 1979 [40]	RIA	n = 24 NL, n = 31 TL	AF	6-keto-PGF_1a_	TL>TNL
					no correlation with GA or cervical dilation
Satoh et al 1979 [41]	RIA	n = 17 serial (8-39wks)	AF	PGFM	TL>TNL
					no pattern with gestation in 3rd TM
		n = 8 TNL, n = 10 TL	plasma		TL>TNL
		n = 53 PTNL			no correlation with GA
					increased with labour stage, peaked at delivery
		n = 30 3rd TM serial	urine	t-PGFM	L>NL
Lewis et al 1980 [42]	GC-MS	n = 6 1st TM	plasma	6-keto-PGF_1a_	2nd-3rd TM > nonpregnant
		n = 9 2nd-3rd TM			
Dubin et al 1981 [43]	RIA	n = 39 serial (16-40wks)	plasma	PGFM	TL>TNL
		n = 17 PTD			no correlation with GA
Sellers et al 1981 [44]	RIA	n = 13 TNL, n = 21 TL	plasma	PGFM	TL>TNL
		n = 12 PTNL, n = 22 PTL			PTL>PTNL
					no difference between PTNL and PTL
					no difference between PTL who delivered term and preterm
					increased with labour stage in PTL and TL
Ylikorkala et al 1981 [45]	RIA	n = 9 serial	plasma	6-keto-PGF_1a_	TL>TNL
					increased with labour stage
Fuchs et al 1982 [46]	RIA	n = 14 TNL, n = 20 TL	plasma	PGFM	TL>TNL
Fuchs et al 1982 [47]	RIA	n = 10 TNL, n = 14 TL	plasma	PGFM	TNL>PTNL
		n = 10 PTNL, n = 15 PTL			PTL>PTNL
Mitchell et al 1982 [48]	RIA	n = 10 TNL, n = 10 TL	plasma	bicyclo-PGEM	TL>TNL
		n = 10 1st TM			1st TM > nonpregnant
		n = 10 2nd TM			decreased in 3rd TM until labour
		n = 10 3rd TM			
Sellers et al 1982 [49]	RIA	n = 10 TL	plasma	PGFM	increased with labour stage, peaked 5min after delivery
Sharma et al 1982 [50]	RIA	n = 92 NL, n = 6 TL	plasma	PGF_2α_	TL>NL
					remained unchanged until 2wks before delivery, then increased
				PGE_2_	no difference between TL and NL
					remained unchanged until 2wks before delivery, then increased
Fuchs et al 1983 [51]	RIA?	n = 4 TNL, n = 17 L	plasma	PGFM	TL>TNL
					increased with labour stage
Nieder & Augustin 1983 [52]	RIA	n = 23 1st TM	AF	PGF, PGE	unchanged from 9-34wks, increase at 35wks
		n = 37 2nd TM			
		n = 103 3rd TM			
Spitz et al 1983 [53]	RIA	n = 12 serial (10-40wks)	plasma	6-keto-PGF_1a_	decrease after 33wks
Husslein & Sinzinger 1984 [54]	RIA	n = 5 TNL, n = 5 TL	plasma	PGEM	TL>TNL
		n = 5 PTNL			no correlation with labour stage
Nagata et al 1984 [55]	RIA	n = 6 term serial	plasma	PGF_2α_	TL>TNL
				PGE_1_, PGE_2_	no difference between NL and L
					no correlation with labour stage
Reddi et al 1984 [56]	RIA	n = 10 TL	AF	PGF, PGFM	increased through 1st stage labour
Sellers et al 1984 [57]	RIA	n = 14 TNL, n = 9 TL	plasma	PGFM	TL>TNL
Yamaguchi & Mori 1984 [58]	RIA	n = 4 <20wks	plasma	PGFM	L>NL
		n = 3 20-30wks			no correlation with GA
		n = 16 30-40wks		6-keto-PGF_1a_	L>NL (nonsignificant)
Brennecke et al 1985 [59]	RIA	n = 9 TNL, n = 27 TL	plasma	PGFM	TL>TNL
		n = 12 serial			increased with labour stage
				bicyclo-PGEM	no difference between TNL and TL
					no correlation with GA or labour stage
Ogino & Jimbo 1986 [60]	RIA	n = 5 24-28wks	plasma	PGF_2α_	peak at 32-36wks
		n = 4 28-32wks		PGE_2_	lowest at 36-40wks
		n = 7 32-36wks			
		n = 8 36-40wks			
Weitz et al 1986 [61]	RIA	n = 6 PTL-TD	plasma	PGFM	PTL>PTNL
		n = 14 PTL-PTD			higher in PTL who delivered PT than those who delivered term
		n = 11 PTNL			
Ylikorkala et al 1986 [62]	RIA	n = 8 TNL, n = 13 TL	urine	6-keto-PGF_1a_	increased with labour stage and with C-section
Berryman et al 1987 [63]	RIA	n = 23 L	AF	PGD_2_	increased through 1st stage labour
Nagata et al 1987 [64]	RIA	n = 9 TL	plasma	PGFM	increased with labour stage (nonsignificant)
				PGE_1_	low throughout labour
Nagata et al 1987 [65]	RIA	n = 7 serial	plasma	PGFM	TL>TNL
					decreased 2wks prior to labour
					increased with labour stage
Romero et al 1987 [66]	RIA	n = 23 PTNL, n = 30 PTL	AF	PGF_2α_, PGE_2_	PTL>PTNL
Noort et al 1988 [67]	RIA	n = 12 1st TM	urine	6-keto-PGF_1a_	L>NL (nonsignificant)
		n = 12 2nd TM			
		n = 12 3rd TM			
		n = 12 TL			
Romero et al 1988 [68]	RIA	n = 32 PTL-TD n = 22 PTL-PTD	AF	PGE_2_	higher in PTL who did not respond to tocolysis than those who responded to tocolysis
Sahmay et al 1988 [69]	RIA	n = 8 TNL, n = 9 TL	AF	PGF_2α_	no difference between TNL and TL
			plasma		TL>TNL
			AF	PGE	no difference between TNL and TL
			plasma		TNL>TL
Noort et al 1989 [70]	RIA	n = 7 TL	plasma	PGFM	increased with labour stage
				6-keto-PGF1a	no correlation with labour stage
Romero et al 1989 [71]	RIA	n = 25 PTL-TD	AF	PGF_2α_	no difference between PTL who delivered term and preterm
		n = 16 PTL-PTD		PGFM, bicyclo-PGEM	higher in PTL who delivered PT than those who responded to tocolysis
Yamamoto & Kitao 1989 [72]	RIA	n = 76 term serial	plasma	PGF_2α_	TL>TNL
					increased with labour stage and delivery
Mazor et al 1990 [73]	RIA	n = 10 PTL-TD	AF	PGF_2α_	no difference between PTL who delivered term and preterm
		n = 10 PTL-PTD		PGE_2_	higher in PTL who delivered PT than those who delivered at term
Norman & Reddi 1990 [74]	RIA	n = 54 TL	AF	PGF_2α_, PGFM, PGE_2_	increased through 1st stage labour
Fairlie et al 1993 [75]	RIA	n = 20 TL	plasma	PGFM	increased with labour stage
				bicyclo-PGEM	in nulliparous: rose after amniotomy but did not change with labour
					in multiparous: rose with amniotomy then increased with labour stage
Hillier et al 1993 [76]	RIA	n = 50 PTL	AF	PGE_2_	high levels associated with PTD and delivery within 1wk of amniocentesis
Johnston et al 1993 [77]	RIA	n = 18 TNL, n = 28 TL	plasma	PGFM	TL>TNL
					rose following amniotomy, then remained constant until delivery
				PGEM	TL>TNL only in primigravid
					rose 1–2 after amniotomy, then remained constant until delivery
MacDonald & Casey 1993 [78]	RIA	n = 50 TNL, n = 190 TL	AF	PGF_2α_	TL>TNL (forebag and upper compartment)
					increased with labour stage, then decreased at 3-5cm dilation
				PGFM	TL>TNL (forebag and upper compartment)
					increased with labour stage, then leveled out at 4–5.5cm dilation until delivery
				PGE_2_	TL>TNL (forebag)
					no difference between TL and TNL in upper compartment
					increased with labour stage, then leveled out at 4–5.5cm dilation until delivery
Romero et al 1993 [79]	RIA	n = 24 NL, n = 16 TL	AF	PGF_2α_, PGFM, PGE_2_, 6-keto-PGF_1α_	TL>NL
Romero et al 1994 [80]	RIA	n = 82 TNL, n = 168 TL	AF	PGF_2α_, PGFM, PGE_2_, 6-keto-PGF_1α_	TL>TNL
					increased through 1st stage labour
Lindsay et al 1995 [81]	ELISA	n = 8 serial (1st-3rd TM)	urine	2,3-dinor-6-keto-PGF_1α_	no correlation with GA
					pregnant >> nonpregnant
Romero et al 1996 [82]	RIA	n = 28 serial (n = 17 L)	AF	PGF_2α_, PGE_2_	TL>TNL
					increased with GA at term
Ichikawa & Minami 1999 [83]	RIA	n = 30 serial	urine	PGF_2α_	TL>NL
					increased from 28-36wks
				PGFM	TL>NL
					increased from 28-36wks and again at 2nd stage of labour
Mitchell et al 2005 [84]	ELISA	n = 24 TNL, n = 37 TL	AF	9α,11β-PGF_2_	TL>TNL
		n = 13 PTNL, n = 56 PTL			PTNL>PTL
Lee et al 2008 [85]	ELISA	n = 68 TNL, n = 34 TL	AF	PGF_2α_	TL>TNL
		n = 65 PTNL			no correlation with GA until 36wks, 25-fold increase at TNL
					increased with labour stage
				PGE_2_	no difference between TL and TNL
					no correlation with GA until 36wks, 2-fold increase at TNL
Lee et al 2009 [86]	ELISA	n = 140 PPROM (n = 126 PTD)	AF	PGF_2α_	high levels associated with low GA at delivery and PTD
Maddipati et al 2014 [87]	LC-MS	n = 10 TNL, n = 35 TL	AF	PGF_2α_, PGFM, PGE_2_, bicyclo-PGEM, PGA_2_, PGJ_2_	TL>TNL
		n = 18 PTNL			
				19-OH-PGE_2_	no difference between TL and TNL
					TNL>PTNL
Park et al 2016 [88]	ELISA	n = 132 PTL (n = 41 PTD)	AF	PGF_2α_	high levels associated with low GA at delivery and PTD
Rosen et al 2019 [89]	GC-NICI-MS	n = 740 (n = 41 sPTD)	urine	PGF_2α_	no difference in 3rd TM levels between term and preterm delivery
Eick et al 2020 [90]	GC-NICI-MS	n = 469 (n = 50 PTD)	urine	PGF_2α_	levels at 20-24wks and 24-28wks higher in preterm than term group
					associated with increased odds of PTB
Peiris et al 2020 [91]	LC-MS	n = 10 TNL, n = 28 TL	AF	PGF_2α_, PGFM, PGE_2_	TL>TNL
Takahashi et al 2021 [92]	LC-MS	n = 11 TNL, n = 10 TL	AF	PGE_2_, 15-keto-PGE_2_, PGEM, 19-OH-PGE_2_	TL>TNL

Abbreviations: TLC = thin layer chromatography, NL = no labour, TL = term labour, LOD = limit of detection, RIA = radioimmunoassay, L = labour, TM = trimester, PTNL = preterm no labour, GC = gas chromatography, AF = amniotic fluid, TNL = term no labour, GC-MS = gas chromatography-mass spectrometry, PGFM = 13,14-dihydro-15-keto-PGF_2α_, t-PGFM = 5α,7α-dihydroxy 11-keto tetranor-prostane 1,16-dioic acid, GA = gestational age, PC = paper chromatography, TD = transmission densitometry, CS = Caesarean section, PTL = preterm labour, PTD = preterm delivery, bicyclo-PGEM = 11-deoxy-13,14-dihydro-15-keto-11,16-bicyclo PGE_2_, PGEM = 13,14-dihydro-15-keto-PGE_2_, PTL-TD = preterm labour-term delivery, PTL-PTD = preterm labour-preterm delivery, PT = preterm, ELISA = enzyme-linked immunosorbent assay, PPROM = preterm premature rupture of membranes, LC-MS = liquid chromatography-mass spectrometry, NICI = negative ion chemical ionization, sPTD = spontaneous preterm delivery.

**Table 3:** Range of prostaglandin concentrations reported using different measurement techniques.

Biofluid	PG/Metabolite	Measurement Technique	Range
plasma	PGE	RIA	NL: 4.8 [36]–3641.2 [69] (pg/ml), L: 5.4 [36]–2429.1 [69] (pg/ml)
	PGF	RIA	NL: 6.2 [36]–480 [29] (pg/ml), L: 7.9 [36]–600 [29] (pg/ml)
	PGE_2_	TLC and biological assay	NL: <200 pg/ml [10]
		RIA	NL: 4.6 [60]–15,600 [55] (pg/ml), L: 559 [50]–21,200 [55] (pg/ml)
	PGE_1_	TLC and biological assay	<200 pg/ml [10]
		RIA	NL: 2600 [55]–10,000 [33] (pg/ml), L: 4500 [64]–6800 [33] (pg/ml)
	PGF_2α_	TLC and biological assay	NL: <200 pg/ml [10], L: <200 [10]– 18,000 [10] (pg/ml)
		GC-MS	NL: <70 [19]–600 [19] (pg/ml), L: <100 [19]–200 [19] (pg/ml)
		RIA	NL: 17 [37]–4600 [55] (pg/ml), L: 33.1 [21]–7900 [55] (pg/ml)
	PGF_1α_	TLC and biological assay	NL: <200 pg/ml [10]
	6-keto-PGF_1α_	GC-MS	NL: 131 [42]–244 [42] (pg/ml)
		RIA	NL: 18.7 [53]–318.6 [58] (pg/ml), L: 21 [70]–608 [70] (pg/ml)
	PGEM	RIA	NL: 58 [54]–554 [77] (pg/ml), L: 82 [54]–433 [77] (pg/ml)
	Bicyclo-PGEM	RIA	NL: 49 [48]–200 [59] (pg/ml), L: 62 [48]–500 [75] (pg/ml)
	PGFM	GC-MS	NL: 31 pg/ml [19], L: 267–942 [19] (pg/ml)
		RIA	NL: 32.1 [61]–490 [41] (pg/ml), L: 20 [75]–2880 [58] (pg/ml)
serum	PGF_2α_	RIA	NL: 200 [13]–1800 [12] (pg/ml), L: 100 [11]–3000 [11] (pg/ml)
AF	PGE	RIA	NL: 89 [37]–1400 [29] (pg/ml), L: 502.8 [69]–8800 [29] (pg/ml)
	PGF	RIA	NL: 50 [27]–1650 [24] (pg/ml), L: 500 [27]–75,000 [27] (pg/ml)
	PGE_2_	GC	NL: <200 [16]–6200 [16] (pg/ml), L: 1200 [16]–17,000 [16] (pg/ml)
		PC and TD	NL: 250 [26]–300 [26] (pg/ml), L: 1700 pg/ml [26]
		RIA	NL: <10 [30]–11,177 [80] (pg/ml), L: 17.8 [76]–28,197 [74] (pg/ml)
		ELISA	NL: 24 [85]–4749 [85] (pg/ml), L: 62 [85]–36,651 [85] (pg/ml)
		LC-MS	NL: <10 [87]–70,493 [87] (pg/ml), L: <10 [87]–105,739 [87] (pg/ml)
	PGE_1_	GC	NL: <500 pg/ml [16], L: <500 pg/ml [16]
		PC and TD	NL: 1000 [26]–1200 [26] (pg/ml), L: 1800 pg/ml [26]
		RIA	NL: <10 [30]–5000 [33] (pg/ml), L: 4400 pg/ml [33]
	PGF_2α_	RIA	NL: 29 [66]–4700 [26] (pg/ml), L: 27 [71]–44,270 [33] (pg/ml)
		ELISA	NL: 8 [85]–926 [85] (pg/ml), L: 78 [85]–15,326 [85] (pg/ml)
		LC-MS	NL: <10 [87]–127 [91] (pg/ml), L: <10 [87]–42,537 [87] (pg/ml)
	PGF_1α_	PC and TD	NL: 1500 [26]–2000 [26] (pg/ml), L: 12,000 pg/ml [26]
	6-keto-PGF_1α_	RIA	NL: 67 [79]–809 [80] (pg/ml), L: 68 [80]–1927 [80] (pg/ml)
	PGEM	LC-MS	NL: 71 pg/ml [92], L: 8425 pg/ml [92]
	Bicyclo-PGEM	RIA	L: 75 [71]- 4275 [71] (pg/ml)
		LC-MS	NL: <10 [87]–66,900 [87] (pg/ml), L: 8361 [87]–133,800 [87] (pg/ml)
	15-keto-PGE_2_	LC-MS	NL: 0 pg/ml [92], L: 210.24 pg/ml [92]
	19-OH-PGE_2_	LC-MS	NL: 0 [92]–221,100 [87] (pg/ml), L: 73.7 [92]–202,675 [87] (pg/ml)
	PGFM	RIA	NL: 80 [79]–1571 [79] (pg/ml), L: 105 [71]–25,028 [56] (pg/ml)
		LC-MS	NL: <10 [87]–114.79 [91] (pg/ml), L: <10 [87]–28,360 [87] (pg/ml)
	PGD_2_	RIA	L: 900 [63]–1800 [63] (pg/ml)
	PGJ_2_	LC-MS	L: 8542.8 pg/ml [87]
	9α,11β-PGF_2_	ELISA	NL: 30 [84]–204 [84] (pg/ml), L: 10 [84]–396 [84] (pg/ml)
	PGA_2_	LC-MS	NL: <10 [87]–16,722 [87] (pg/ml), L: <10 [87]–50,167 [87] (pg/ml)
urine	PGF_2α_	RIA	NL: 0.99 [83]–1.85 [83] (pg/g creatinine), L: 2.03 [83]–3.14 [83] (pg/g creatinine)
		GC-NICI-MS	NL: 1840 [90]–2060 [89] (pg/ml)
	6-keto-PGF_1α_	RIA	NL: 114,000 [67]–571,000 [67] (pg/g creatinine), L: 426,980 [62]–1,219,000 [67] (pg/g creatinine)
	PGFM	RIA	NL: 1.82 [83]–4.87 [83] (pg/g creatinine), L: 7.93 [83]–12.70 [83] (pg/g creatinine)
	t-PGFM	RIA	NL: 0.46 [20]–2.32 [20] (μg/hr), L: 1.06 [41]–2.50 [31] (μg/hr)
	2,3-dinor-6-keto-PGF_1α_	ELISA	NL: 623,232 [81]–1,096,181 [81] (pg/ml)

Published data presented as ng/ml, nanomolars, or picomolars were converted to pg/ml and data presented as ng/g creatinine or ng/mmol creatinine were converted to pg/g creatinine. Data published in μg/hr were not converted and are presented as in the original article.

Abbreviations: RIA = radioimmunoassay, NL = non-labour, L = labour, TLC = thin layer chromatography, GC-MS = gas chromatography-mass spectrometry, PGEM = 13,14-dihydro-15-keto-PGE_2_, bicyclo-PGEM = 11-deoxy-13,14-dihydro-15-keto-11,16-bicyclo PGE_2_, PGFM = 13,14-dihydro-15-keto-PGF_2α_, PC = paper chromatography, TD = transmission densitometry, ELISA = enzyme-linked immunosorbent assay, LC-MS = liquid chromatography-mass spectrometry, GC-NICI-MS = gas chromatography-negative ion chemical ionization-mass spectrometry, t-PGFM = 5α,7α-dihydroxy 11-keto tetranor-prostane 1,16-dioic acid.

### Amniotic fluid

#### Labour and non-labour

In total, 25 studies compared amniotic fluid prostaglandins in labour vs non-labour. PGF_2α_ increased in labouring participants compared to non-labouring participants in most studies, (Table 2) however, one study found no difference [69]. Similarly, PGE_2_ was reported to increase with labour in 11/12 studies [16, 26, 30, 66, 78–80, 82, 87, 91, 92]. 6-keto-PGF_1α_ and PGFM and were reported to increase in labouring participants compared to non-labouring participants in three [40, 79, 80] and seven [32, 41, 78–80, 87, 91] studies, respectively. Results were mixed for PGE_1_ [26, 33]. PGE was not found to increase with labour [29, 69].

#### Prior to labour onset

Of the included amniotic fluid studies, 15 measured prostaglandins at more than one time point throughout pregnancy. PGF_2α_, PGF_1α_, and PGF were generally found to increase around term or prior to labour [17, 22, 27, 28, 30, 37, 52, 82, 85], though two studies found no pattern throughout pregnancy [26, 33]. Among studies that measured PGE or PGE_2_, most (4/6) reported increased levels around 35–36 weeks [30, 37, 52, 85]. PGE_1_ was not found to change with gestational age [26, 30, 33].

#### Predicting preterm labour

Six amniotic fluid studies investigated prostaglandins as predictors of preterm labour. Some studies suggest that PGF_2α_ may be predictive of preterm delivery in those with threatened preterm labour [88] and PPROM [86] however, results are mixed [71, 73]. PGFM and bicyclo-PGEM were found in higher levels in participants with preterm labour leading to preterm delivery compared to those who eventually delivered at term [71]. Increased PGE_2_ levels may be predictive of delivery before term [68, 73] and before 34 weeks [76].

### Blood

#### Labour and non-labour

In total, 27 studies compared labour and non-labour groups. PGF_2α_ was reported to increase with labour compared to non-labour in most (6/8) studies [21, 41, 50, 55, 69, 72]. All 15 studies that measured PGFM reported higher levels in labour compared to non-labour (Table 2). Three studies measuring PGE reported varying results [29, 36, 69]. PGE_1_, PGE_2_, and PGF were all generally found to remain unchanged with labour [29, 33, 36, 50, 55].

#### Prior to labour onset

In total, 18 studies obtained measurements of plasma throughout pregnancy. Among those that measured PGF_2α_, some found increasing levels at or near term [37, 50, 60] however results were conflicting [21, 28, 33]. In 5/6 studies PGFM was not found to change with increasing gestational age [39, 41, 43, 44, 58]. Results for PGF_2α_ in serum were mixed [11–13].

#### Predicting preterm labour

Two studies investigated prostaglandins in plasma as predictors of preterm labour. One study found that PGFM levels were higher in participants in preterm labour who delivered preterm compared to those who went on to deliver at term [61] though the other study reported no significant difference [44].

### Urine

#### Labour and non-labour

Five studies compared labouring vs non-labouring groups. The metabolite t-PGFM was reported to increase with labour compared to non-labour [20, 31, 41].

#### Prior to labour onset

Four studies measured changes in prostaglandins in urine throughout pregnancy. PGF_2α_, PGFM, and t-PGFM were reported to increase around term, though this was only reported in one study for each prostaglandin/metabolite [20, 83]. The metabolite 2,3-dinor-6-keto-PGF_1α_ did not change throughout pregnancy [81].

#### Predicting preterm labour

Two urine studies investigated prostaglandins as predictors of preterm labour. One found that PGF_2α_ levels in urine samples collected at median 32.1 weeks were not significantly different between participants who delivered at term and those that delivered preterm [89]. In contrast, averaged levels of PGF_2α_ in urine were also found to be associated with increased odds of preterm birth (OR = 1.98) [90].

### Serial prostaglandin measurement during spontaneous labour

Although not a primary study question of this review, we noted that n = 40 studies obtained serial samples during labour. In general, prostaglandins measured in amniotic fluid increased throughout labour. Results were mixed for those that measured plasma and serum.

### Quality assessment

Scores from the quality assessment were distributed as follows: 17% scored between 0–3, 41% scored between 4–6, and 42% scored between 7–9. The areas with the lowest scores were researcher blinding and sufficiency of sample number for internal validity. Scores for each study can be found in S3 Table.

## Discussion

We demonstrate, through a systematic review of the literature investigating prostaglandins and metabolites in peripheral biofluids during pregnancy and labour, that prostaglandins of the PGE and PGF families do exhibit changes through pregnancy and labour, though results are inconsistent and inconclusive. Changes in PGE_2_, PGF_2α_, and PGFM levels with labour are most prominent in amniotic fluid, and to a lesser extent in blood. Similarly, our synthesis suggests that PGE_2_, PGF_2α_ and PGF_1α_ increase in amniotic fluid as pregnancy progresses and peak around term, though in plasma, a consistent pattern is unclear. Patterns in urine prostaglandin levels were inconclusive due to a relatively small number of studies investigating this biofluid. An important limitation is a general lack of data on prostaglandins and metabolites outside the PGE and PGF families, and as such we are unable to comment on their potential role in pregnancy and labour. Further, few studies examined prostaglandins as biomarkers for preterm labour and more research is needed to provide conclusive evidence for which prostaglandins or metabolites examined could offer the best options for prediction.

### Measurement techniques for prostaglandins

Inconsistent study designs and methods greatly limited our ability to compare findings across studies. Up to the late 1990’s, researchers most commonly used radioimmunoassay techniques, which can be highly sensitive, but are often limited by the specificity of the antibody used and the potential of antibody cross-reactivity with similar molecules [87]. One study included in this review developed and reported on a radioimmunoassay for PGF_2α_ with a cross-reactivity with PGF_1α_ of 12.2% [17], which may have obscured patterns in PGF_2α_ and made it difficult to ascertain fine-tuning of the prostaglandin pathway among similar molecules. Furthermore, multiple other studies using radioimmunoassay techniques were unable to differentiate between PGF_2α_ and PGF_1α_, and PGE_2_ and PGE_1_ and therefore could only report on levels of PGF and PGE, respectively, making it difficult to compare the results of these studies with others. Lack of specificity and accuracy in these radioimmunoassay techniques may have contributed to the discrepancies across results and highlights the importance of re-visiting dogma in light of novel evidence and technologies. In contrast, the high specificity and sensitivity of mass spectrometry for lipid identification suggests that this method may be more suitable and accurate for measurement of prostaglandins [87]. Additionally, the capability of mass spectrometry to co-assess multiple prostaglandins and metabolites can provide a quantitative profile of prostaglandins before and during labour, as well as identify prostaglandins and/or metabolites not previously measured that may play a role in pregnancy and/or labour [93].

### Considerations among unique biofluids

Among the studies included in this review, the most assayed biofluid was maternal plasma. Although an appealing fluid due to its ability to be sampled relatively easily, results from measurements in plasma were often conflicting, especially among studies that measured primary prostaglandins. Accurate measurement of changes in primary prostaglandin levels in blood is complicated by their rapid metabolism and correspondingly short half-life [94, 95]. This difficulty is further compounded by the production of prostaglandins by platelets that occurs during isolation of plasma and storage of samples [36, 96, 97]. Measurement of plasma PGFM appears to be a good alternative for PGF_2α_, as there is no evidence that this metabolite is formed during sample collection or isolation and therefore may more accurately reflect endogenous prostaglandin production [19]. The primary metabolite of PGE_2_, however, is chemically unstable [98], which necessitates the measurement of its degradation product, bicyclo-PGEM, for an accurate index of PGE_2_ production [99]. Therefore, results from early studies measuring primary prostaglandins and/or PGEM in plasma and/or serum should be interpreted with these considerations in mind and future studies in blood should aim to measure PGFM or bicyclo-PGEM as indices of PGF_2α_ or PGE_2_ production, respectively.

Amniotic fluid lacks prostaglandin metabolizing enzymes [100, 101], which suggests that measurement of the primary prostaglandins in this fluid may be more accurate than in serum or plasma. However, sampling amniotic fluid is more difficult and may introduce infections harmful to the developing fetus, making this fluid impractical as a predictive resource. Additionally, prostaglandin levels vary based on method of collection and region of the amniotic sac [102, 103] which complicates any interpretation of results from studies and limits the clinical utility of an amniotic fluid test for prediction of preterm labour.

Measurement of the main urinary metabolite of PGF_2α_ may be preferable to measuring PGFM in plasma or serum in some cases, as a significant portion of circulating PGF_2α_ is eventually excreted into the urine [104]. In the present investigation, we identified only nine studies that assayed urine, and we suggest that the presence of urinary metabolites of prostaglandins during pregnancy and labour merits further study.

### Demographic and clinical information

Among the articles included in this review, we noted that very few provided complete demographic and clinical information on their participants. Factors including age, race/ethnicity, membrane status, and gravidity/parity may impact prostaglandins levels and a lack of consideration for these variables may obscure patterns of prostaglandin levels throughout pregnancy and labour. Complete descriptions of gestational age groups and clearly defined outcomes for both term and preterm labour would additionally make studies more easily comparable. As well, preterm labour is generally defined as labour occurring before 37 weeks gestation, however the pathophysiological processes involved in extreme preterm birth (<28 weeks) may vary dramatically from those near term [105]. Therefore, stratification of outcome groups based on gestational age at delivery may be more informative, though would require larger sample sizes to maintain statistical power.

### Role for other prostaglandins

While prostaglandins of the E and F series are most clinically targeted for labour management, there is evidence to suggest that other members of the prostaglandin family may play a role in pregnancy and labour. For example, PGD_2_ has been shown to increase uterine contractility and blood flow in various mammals [106–108] and is associated with cervical dilation in humans [63]. Two metabolites of PGD_2_, 9α,11β-PGF_2_ and PGJ_2_, were each identified only once among the articles included in this review and were both reported to increase with term labour [84, 87]. These metabolites may be of interest to future researchers, as the development of new methodologies such as mass spectrometry have allowed for more accurate and sensitive measurements of select members of the prostaglandin pathway.

### Limitations

The main limitation of this review is that only studies in English or with an available English translation were included, which may have resulted in missing some relevant articles. However, current resources limited the feasibility of including non-English studies.

## Conclusion

We have identified evidence to suggest that prostaglandin levels, particularly within the PGE and PGF families, do increase in some biofluids during pregnancy and labour. However, changing prostaglandin levels throughout pregnancy and labour are likely highly complex and warrant further investigation, including serial measurements with more precise methodologies in higher-powered studies. Two important limitations identified in this review are the lack of data on the complexity of the prostaglandin pathway outside of the PGE and PGF families and the inherent difficulty in measuring primary prostaglandins in blood, due to their short half-lives in this biofluid. With the advent of i) new methodologies that can assess multiple prostaglandins and metabolites together, ii) a more developed understanding of the range of prostaglandins and iii) a better understanding of the heterogeneous nature of term and preterm labour, future studies that take each of these parameters into account in their study design will help provide further insight into the changing levels of prostaglandins in pregnancy and labour.

## Supporting information

S1 Table EMBASE search terms. (DOCX)

S2 Table MEDLINE search terms. (DOCX)

S3 Table Quality assessment scores. (DOCX)

S1 Checklist PRISMA 2020 checklist. (DOC)

## References

[1] Challis JR, Sloboda DM, Alfaidy N, Lye SJ, Gibb W, Patel FA, et al. Prostaglandins and mechanisms of preterm birth. Reproduction. 2002;124(1):1–17. doi: 10.1530/rep.0.1240001 12090913

[2] Urrego D, Liwa AC, Cole WC, Wood SL, Slater DM. Cyclooxygenase inhibitors for treating preterm labour: What is the molecular evidence? Can J Physiol Pharmacol. 2019;97(3):222–31. doi: 10.1139/cjpp-2018-0380 30661374

[3] Green K. Determination of prostaglandins in body fluids and tissues. Acta Obstet Gynecol Scand Suppl. 1979;87:15–20. 288293

[4] Olson DM. The role of prostaglandins in the initiation of parturition. Best Pract Res Clin Obstet Gynaecol. 2003;17(5):717–30. doi: 10.1016/s1521-6934(03)00069-5 12972010

[5] Reinebrant HE, Pileggi-Castro C, Romero CLT, Dos Santos RAN, Kumar S, Souza JP, et al. Cyclo-oxygenase (COX) inhibitors for treating preterm labour. Cochrane Database Syst Rev. 2015;2015(6):CD001992–CD. doi: 10.1002/14651858.CD001992.pub3 26042617 PMC7068172

[6] Schlembach D, Maner WL, Garfield RE, Maul H. Monitoring the progress of pregnancy and labor using electromyography. Eur J Obstet Gynecol Reprod Biol. 2009;144 Suppl 1:S33–9. doi: 10.1016/j.ejogrb.2009.02.016 19278772

[7] Heng YJ, Liong S, Permezel M, Rice GE, Di Quinzio MK, Georgiou HM. The interplay of the interleukin 1 system in pregnancy and labor. Reprod Sci. 2014;21(1):122–30. doi: 10.1177/1933719113492204 23749763 PMC3857767

[8] Vogel JP, Chawanpaiboon S, Moller AB, Watananirun K, Bonet M, Lumbiganon P. The global epidemiology of preterm birth. Best Pract Res Clin Obstet Gynaecol. 2018;52:3–12. doi: 10.1016/j.bpobgyn.2018.04.003 29779863

[9] Hadley EE, Richardson LS, Torloni MR, Menon R. Gestational tissue inflammatory biomarkers at term labor: A systematic review of literature. Am J Reprod Immunol. 2018;79(2): doi: 10.1111/aji.12776 29076197 PMC5783758

[10] Karim SM. Appearance of prostaglandin F2-alpha in human blood during labour. Br Med J. 1968;4(5631):618–21. doi: 10.1136/bmj.4.5631.618 5723366 PMC1912474

[11] Brummer HC. Serum PGF2 alpha levels during late pregnancy, labour and the puerperium. Prostaglandins. 1972;2(3):185–94. doi: 10.1016/s0090-6980(72)80022-4 4653733

[12] Gutierrez-Cernosek RM, Zuckerman J, Levine L. Prostaglandin F2alpha levels in sera during human pregnancy. Prostaglandins. 1972;1(4):331–7. doi: 10.1016/s0090-6980(72)80010-8 4676641

[13] Brummer HC. Serum PGF2alpha levels during human pregnancy. Prostaglandins. 1973;3(1):3–5. doi: 10.1016/0090-6980(73)90132-9 4693718

[14] Brummer HC, Craft IL. Prostaglandin F2alpha and labour. Acta Obstet Gynecol Scand. 1973;52(3):273–5. doi: 10.3109/00016347309158326 4743782

[15] Hertelendy F, Woods R, Jaffe BM. Prostaglandin E levels in peripheral blood during labor. Prostaglandins. 1973;3(2):223–7. doi: 10.1016/0090-6980(73)90090-7 4729571

[16] Keirse MJ, Turnbull AC. E prostaglandins in amniotic fluid during late pregnancy and labour. J Obstet Gynaecol Br Commonw. 1973;80(11):970–3. doi: 10.1111/j.1471-0528.1973.tb02958.x 4752988

[17] Salmon JA, Amy J-J. Levels of prostaglandin F2α in amniotic fluid during pregnancy and labour. Prostaglandins. 1973;4(4):523–33.

[18] Challis JR, Osathanondh R, Ryan KJ, Tulchinsky D. Maternal and fetal plasma prostaglandin levels at vaginal delivery and cesarean section. Prostaglandins. 1974;6(4):281–8. doi: 10.1016/s0090-6980(74)80002-x 4853104

[19] Green K, Bygdeman M, Toppozada M, Wiqvist N. The role of prostaglandin F2alpha in human parturition. Endogenous plasma levels of 15-keto-13,14-dihydro-prostaglandin F2alpha during labor. Am J Obstet Gynecol. 1974;120(1):25–31. 4846168

[20] Hamberg M. Quantitative studies on prostaglandin synthesis in man III. Excretion of the major urinary metabolite of prostaglandins F1α and F2α during pregnancy. Life Sci. 1974;14(2):247–52. doi: 10.1016/0024-3205(74)90054-x 4813589

[21] Hennam JF, Johnson DA, Newton JR, Collins WP. Radioimmunoassay of prostaglandin F-2-alpha in peripheral venous plasma from men and women. Prostaglandins. 1974;5(6):531–42. doi: 10.1016/s0090-6980(74)80028-6 4856639

[22] Hibbard BM, Sharma SC, Fitzpatrick RJ, Hamlett JD. Prostaglandin F2alpha concentrations in amniotic fluid in late pregnancy. J Obstet Gynaecol Br Commonw. 1974;81(1):35–8. doi: 10.1111/j.1471-0528.1974.tb00361.x 4818314

[23] Hillier K, Calder AA, Embrey MP. Concentrations of prostaglandin F2alpha in amniotic fluid and plasma in spontaneous and induced labours. The Journal of obstetrics and gynaecology of the British Commonwealth. 1974;81(4):257–63. doi: 10.1111/j.1471-0528.1974.tb00457.x 4824681

[24] Keirse MJ, Flint AP, Turnbull AC. F prostaglandins in amniotic fluid during pregnancy and labour. J Obstet Gynaecol Br Commonw. 1974;81(2):131–5. doi: 10.1111/j.1471-0528.1974.tb00431.x 4816023

[25] MacDonald PC, Schultz FM, Duenhoelter JH, Gant NF, Jimenez JM, Pritchard JA, et al. Initiation of human parturition. I. Mechanism of action of arachidonic acid. Obstet Gynecol. 1974;44(5):629–36. 4420973

[26] Singh EJ, Zuspan FP. Content of amniotic fluid prostaglandins in normal, diabetic, and drug-abuse human pregnancy. Am J Obstet Gynecol. 1974;118(3):358–61. doi: 10.1016/s0002-9378(16)33793-0 4810040

[27] Hillier K, Calder AA, MacKenzie IZ. Proceedings: Prostaglandin F alpha in amniotic fluid in man. J Endocrinol. 1975;64(1):13P. 1167892

[28] Johnson DA, Manning PA, Hennam JF, Newton JR, Collins WP. The concentration of prostaglandin f2alpha in maternal plasma, foetal plasma and amniotic fluid during pregnancy in women. Acta Endocrinol. 1975;79(3):589–97. doi: 10.1530/acta.0.0790589 1173509

[29] Pokoly TB, Jordan VC. Relation of steroids and prostaglandin at vaginal delivery and cesarean section. Obstet Gynecol. 1975;46(5):577–80. 1196562

[30] Dray F, Frydman R. Primary prostaglandins in amniotic fluid in pregnancy and spontaneous labor. Am J Obstet Gynecol. 1976;126(1):13–9. doi: 10.1016/0002-9378(76)90457-9 961739

[31] Granstrom E, Kindahl H. Radioimmunoassay for urinary metabolites of prostaglandin F2alpha. Prostaglandins. 1976;12(5):759–83. doi: 10.1016/0090-6980(76)90051-4 981701

[32] Keirse MJ, Mitchell MD, Turnbull AC. Changes in prostaglandin F and 13,14-dihydro-15-keto-prostaglandin F concentrations in amniotic fluid at the onset of and during labour. Br J Obstet Gynaecol. 1977;84(10):743–6. doi: 10.1111/j.1471-0528.1977.tb12484.x 921911

[33] Kinoshita K, Satoh K, Sakamoto S. Prostaglandin F2alpha and E1 in plasma and amniotic fluid during human pregnancy and labor. Endocrinol Jpn. 1977;24(2):155–62. doi: 10.1507/endocrj1954.24.155 872820

[34] TambyRaja RL, Salmon JA, Karim SM, Ratnam SS. F prostaglandin levels in amniotic fluid in premature labour. Prostaglandins. 1977;13(2):339–48. doi: 10.1016/0090-6980(77)90013-2 847237

[35] Haning RV Jr., Barrett DA, Alberino SP, Lynskey MT, Donabedian R, Speroff L. Interrelationships between maternal and cord prolactin, progesterone, estradiol, 13,14-dihydro-15-keto-prostaglandin F2alpha, and cord cortisol at delivery with respect to initiation of parturition. Am J Obstet Gynecol. 1978;130(2):204–10. doi: 10.1016/0002-9378(78)90367-8 563675

[36] Mitchell MD, Flint AP, Bibby J, Brunt J, Arnold JM, Anderson AB, et al. Plasma concentrations of prostaglandins during late human pregnancy: influence of normal and preterm labor. J Clin Endocrinol Metab. 1978;46(6):947–51. doi: 10.1210/jcem-46-6-947 263475

[37] Nieder J, Augustin W. Investigations on prostaglandin levels in amniotic fluid and maternal blood during late pregnancy. Acta Biol Med Ger. 1978;37(5–6):945–7. 742318

[38] Zuckerman H, Reiss U, Atad J, Lampert I, Ezra SB, Sklan D. Prostaglandin F2alpha human blood during labor. Obstet Gynecol. 1978;51(3):311–4. doi: 10.1097/00006250-197803000-00011 628533

[39] Ghodgaonkar RB, Dubin NH, Blake DA, King TM. 13, 14-dihydro-15-keto-prostaglandin F2alpha concentrations in human plasma and amniotic fluid. Am J Obstet Gynecol. 1979;134(3):265–9. doi: 10.1016/s0002-9378(16)33031-9 453260

[40] Mitchell MD, Keirse MJ, Brunt JD, Anderson AB, Turnbull AC. Concentrations of the prostacyclin metabolite, 6-keto-prostaglandin F1 alpha, in amniotic fluid during late pregnancy and labour. Br J Obstet Gynaecol. 1979;86(5):350–3. doi: 10.1111/j.1471-0528.1979.tb10609.x 380629

[41] Satoh K, Yasumizu T, Fukuoka H, Kinoshita K, Kaneko Y, Tsuchiya M, et al. Prostaglandin F2 alpha metabolite levels in plasma, amniotic fluid, and urine during pregnancy and labor. Am J Obstet Gynecol. 1979;133(8):886–90. doi: 10.1016/0002-9378(79)90306-5 434032

[42] Lewis PJ, Boylan P, Friedman LA, Hensby CN, Downing I. Prostacyclin in pregnancy. Br Med J. 1980;280(6231):1581–2. doi: 10.1136/bmj.280.6231.1581 7000245 PMC1601850

[43] Dubin NH, Johnson JW, Calhoun S, Ghodgaonkar RB, Beck JC. Plasma prostaglandin in pregnant women with term and preterm deliveries. Obstet Gynecol. 1981;57(2):203–6. 7465125

[44] Sellers SM, Mitchell MD, Bibby JG, Anderson AB, Turnbull AC. A comparison of plasma prostaglandin levels in term and preterm labour. Br J Obstet Gynaecol. 1981;88(4):362–6. doi: 10.1111/j.1471-0528.1981.tb00997.x 7225293

[45] Ylikorkala O, Makarainen L, Viinikka L. Prostacyclin production increases during human parturition. Br J Obstet Gynaecol. 1981;88(5):513–6. doi: 10.1111/j.1471-0528.1981.tb01025.x 7016168

[46] Fuchs A-R, Husslein P, Sumulong L, Fuchs F. The origin of circulating 13,14-dihydro-15-keto-prostaglandin F2α during delivery. Prostaglandins. 1982;24(5):715–22. doi: 10.1016/0090-6980(82)90039-9 7163513

[47] Fuchs AR, Husslein P, Sumulong L, Micha JP, Dawood MY, Fuchs F. Plasma levels of oxytocin and 13, 14-dihydro-15-keto prostaglandin F2 alpha in preterm labor and the effect of ethanol and ritodrine. Am J Obstet Gynecol. 1982;144(7):753–9. doi: 10.1016/0002-9378(82)90347-7 7148897

[48] Mitchell MD, Ebenhack K, Kraemer DL, Cox K, Cutrer S, Strickland DM. A sensitive radioimmunoassay for 11-deoxy-13, 14-dihydro-15-keto-11, 16-cyclo-prostaglandin E2: application as an index of prostaglandin E2 biosynthesis during human pregnancy and parturition. Prostaglandins Leukot Med. 1982;9(5):549–57. doi: 10.1016/0262-1746(82)90036-1 6960378

[49] Sellers SM, Hodgson HT, Mitchell MD, Anderson ABM, Turnbull AC. Raised prostaglandin levels in the third stage of labor. Am J Obstet Gynecol. 1982;144(2):209–12. doi: 10.1016/0002-9378(82)90629-9 7114131

[50] Sharma SC, Walzman M, Molloy A, Bonnar J. Relationship of total ascorbic acid to prostaglandins F2 alpha and E2 levels in the blood of women during the 3rd trimester of normal pregnancy. Int J Vitam Nutr Res. 1982;52(3):312–9. 6959982

[51] Fuchs AR, Goeschen K, Husslein P, Rasmussen AB, Fuchs F. Oxytocin and the initiation of human parturition III. Plasma concentrations of oxytocin and 13,14-dihydro-15-keto-prostaglandin F2alpha in spontaneous and oxytocin-induced labor at term. Am J Obstet Gynecol. 1983;147(5):497–502. 6638091

[52] Nieder J, Augustin W. Increase of prostaglandin E and F equivalents in amniotic fluid during late pregnancy and rapid PG F elevation after cervical dilatation. Prostaglandins Leukot Med. 1983;12(3):289–97. doi: 10.1016/0262-1746(83)90007-0 6581485

[53] Spitz B, Deckmyn H, Van Assche FA, Vermylen J. Prostacyclin production in whole blood throughout normal pregnancy. Clin Exp Hypertens B. 1983;2(2):191–202. doi: 10.3109/10641958309006079 6347442

[54] Husslein P, Sinzinger H. Concentration of 13,14-dihydro-15-keto-prostaglandin E2 in the maternal peripheral plasma during labour of spontaneous onset. Br J Obstet Gynaecol. 1984;91(3):228–31. doi: 10.1111/j.1471-0528.1984.tb04757.x 6704346

[55] Nagata I, Kato K, Makimura N, Furuya K. Differences in human plasma oxytocin, prostaglandin E1, E2 and F2 alpha levels between spontaneous and amniotomy-induced labors. Asia Oceania J Obstet Gynaecol. 1984;10(4):513–20. doi: 10.1111/j.1447-0756.1984.tb00719.x 6598029

[56] Reddi K, Kambaran SR, Norman RJ, Joubert SM, Philpott RH. Abnormal concentrations of prostaglandins in amniotic fluid during delayed labour in multigravid patients. Br J Obstet Gynaecol. 1984;91(8):781–7. doi: 10.1111/j.1471-0528.1984.tb04850.x 6590093

[57] Sellers SM, Mitchell MD, Anderson AB, Turnbull AC. The influence of spontaneous and induced labour on the rise in prostaglandins at amniotomy. Br J Obstet Gynaecol. 1984;91(9):849–52. doi: 10.1111/j.1471-0528.1984.tb03695.x 6591948

[58] Yamaguchi M, Mori N. 6-Keto prostaglandin F1 alpha, thromboxane B2 and 13,14-dihydro-15-keto prostaglandin F levels in maternal and fetal plasma. Prostaglandins Leukot Med. 1984;15(3):325–36. doi: 10.1016/0262-1746(84)90132-x 6593746

[59] Brennecke SP, Castle BM, Demers LM, Turnbull AC. Maternal plasma prostaglandin E2 metabolite levels during human pregnancy and parturition. Br J Obstet Gynaecol. 1985;92(4):345–9. doi: 10.1111/j.1471-0528.1985.tb01107.x 3857076

[60] Ogino M, Jimbo T. Comparison of plasma prostaglandins in the non-pregnant and pregnant women: a longitudinal study. Nihon Sanka Fujinka Gakkai Zasshi. 1986;38(7):1120–4. 3462267

[61] Weitz CM, Ghodgaonkar RB, Dubin NH, Niebyl JR. Prostaglandin F metabolite concentration as a prognostic factor in preterm labor. Obstet Gynecol. 1986;67(4):496–9. 3457328

[62] Ylikorkala O, Paatero H, Suhonen L, Viinikka L. Vaginal and abdominal delivery increases maternal urinary 6-keto-prostaglandin F(1alpha) excretion. Br J Obstet Gynaecol. 1986;93(9):950–4. doi: 10.1111/j.1471-0528.1986.tb08014.x 3768289

[63] Berryman GK, Strickland DM, Hankins GD, Mitchell MD. Amniotic fluid prostaglandin D2 in spontaneous and augmented labor. Life Sci. 1987;41(13):1611–4. doi: 10.1016/0024-3205(87)90728-4 3476816

[64] Nagata I, Seki K, Uesato T. Changes in plasma oxytocin, prostaglandin E1, and 13,14-dihydro-15-keto-prostaglandin F(2alpha) during labor induced by prostaglandin E2 or F(2alpha) and spontaneous labor. Acta Obstet Gynaecol Jpn. 1987;39(9):1627–33. 3479503

[65] Nagata I, Sunaga H, Furuya K, Makimura N, Kato K. Changes in the plasma prostaglandin F2 alpha metabolite before and during spontaneous labor and labor induced by amniotomy, oxytocin and prostaglandin E2. Endocrinol Jpn. 1987;34(2):153–9. doi: 10.1507/endocrj1954.34.153 3476296

[66] Romero R, Emamian M, Wan M, Quintero R, Hobbins JC, Mitchell MD. Prostaglandin concentrations in amniotic fluid of women with intra-amniotic infection and preterm labor. Am J Obstet Gynecol. 1987;157(6):1461–7. doi: 10.1016/s0002-9378(87)80245-4 3480691

[67] Noort WA, De Zwart FA, Keirse MJNC. Changes in urinary 6-keto-prostaglandin F1(alpha) excretion during pregnancy and labor. Prostaglandins. 1988;35(4):573–82. doi: 10.1016/0090-6980(88)90032-9 3247472

[68] Romero R, Wu YK, Mazor M, Hobbins JC, Mitchell MD. Amniotic fluid prostaglandin E2 in preterm labor. Prostaglandins Leukot Essent Fatty Acids. 1988;34(3):141–5. doi: 10.1016/0952-3278(88)90137-8 3222272

[69] Sahmay S, Coke A, Hekim N, Atasu T. Maternal, umbilical, uterine and amniotic prostaglandin E and F2 alpha levels in labour. J Int Med Res. 1988;16(4):280–5. doi: 10.1177/030006058801600405 3169373

[70] Noort WA, van Bulck B, Vereecken A, de Zwart FA, Keirse MJNC. Changes in plasma of PGF2α and PGI2 metabolites at and after delivery at term. Prostaglandins. 1989;37(1):3–12. doi: 10.1016/0090-6980(89)90027-0 2655010

[71] Romero R, Wu YK, Sirtori M, Oyarzun E, Mazor M, Hobbins JC, et al. Amniotic fluid concentrations of prostaglandin F2 alpha, 13,14-dihydro-15-keto-prostaglandin F2 alpha (PGFM) and 11-deoxy-13,14-dihydro-15-keto-11, 16-cyclo-prostaglandin E2 (PGEM-LL) in preterm labor. Prostaglandins. 1989;37(1):149–61. doi: 10.1016/0090-6980(89)90038-5 2717777

[72] Yamamoto K, Kitao M. Interrelation among endogenous catecholamines, prostaglandin F2 alpha and prolactin in last trimester and during parturition. Nippon Sanka Fujinka Gakkai zasshi. 1989;41(9):1479–86. 2555427

[73] Mazor M, Wiznitzer A, Maymon E, Leiberman JR, Cohen A. Changes in amniotic fluid concentrations of prostaglandins E2 and F2 alpha in women with preterm labor. Isr J Med Sci. 1990;26(8):425–8. 2401604

[74] Norman RJ, Reddi K. Prostaglandins in dysfunctional labour; evidence for altered production of prostaglandin F2 alpha. Reprod Fertil Dev. 1990;2(5):563–74. doi: 10.1071/rd9900563 2127461

[75] Fairlie F, Phillips G, McLaren M, Calder A, Walker J. Uterine activity in spontaneous labour and maternal peripheral plasma prostaglandin E2 and F(2alpha) metabolites. J Perinat Med. 1993;21(1):35–42. doi: 10.1515/jpme.1993.21.1.35 8487148

[76] Hillier SL, Witkin SS, Krohn MA, Watts DH, Kiviat NB, Eschenbach DA. The relationship of amniotic fluid cytokines and preterm delivery, amniotic fluid infection, histologic chorioamnionitis, and chorioamnion infection. Obstet Gynecol. 1993;81(6):941–8. 8497360

[77] Johnston TA, Greer IA, Kelly RW, Calder AA. Plasma prostaglandin metabolite concentrations in normal and dysfunctional labour. Br J Obstet Gynaecol. 1993;100(5):483–8. doi: 10.1111/j.1471-0528.1993.tb15277.x 8518251

[78] MacDonald PC, Casey ML. The accumulation of prostaglandins (PG) in amniotic fluid is an aftereffect of labor and not indicative of a role for PGE2 or PGF2 alpha in the initiation of human parturition. J Clin Endocrinol Metab. 1993;76(5):1332–9. doi: 10.1210/jcem.76.5.8496326 8496326

[79] Romero R, Baumann P, Gomez R, Salafia C, Rittenhouse L, Barberio D, et al. The relationship between spontaneous rupture of membranes, labor, and microbial invasion of the amniotic cavity and amniotic fluid concentrations of prostaglandins and thromboxane B2 in term pregnancy. Am J Obstet Gynecol. 1993;168(6 Pt 1):1654–8. doi: 10.1016/0002-9378(93)90675-9 8317506

[80] Romero R, Baumann P, Gonzalez R, Gomez R, Rittenhouse L, Behnke E, et al. Amniotic fluid prostanoid concentrations increase early during the course of spontaneous labor at term. Am J Obstet Gynecol. 1994;171(6):1613–20. doi: 10.1016/0002-9378(94)90412-x 7802078

[81] Lindsay C, Moutquin JM, Gaudreault CR, Forest JC. Development of an enzyme-linked immunosorbent assay for 2,3-dinor-6-keto-prostaglandin F(1alpha) in urine using a monoclonal antibody. Clin Biochem. 1995;28(4):395–400. doi: 10.1016/0009-9120(95)00018-5 8521593

[82] Romero R, Munoz H, Gomez R, Parra M, Polanco M, Valverde V, et al. Increase in prostaglandin bioavailability precedes the onset of human parturition. Prostaglandins Leukot Essent Fatty Acids. 1996;54(3):187–91. doi: 10.1016/s0952-3278(96)90015-0 8860106

[83] Ichikawa M, Minami M. Correlations between corticotropin-releasing hormone and L-3,4-dihydroxyphenylalanine in plasma, and dopamine and prostaglandins in urine in pregnant mothers. Biog Amines. 1999;15(2):287–306.

[84] Mitchell MD, Chang MC, Chaiworapongsa T, Lan H-Y, Helliwell RJA, Romero R, et al. Identification of 9alpha,11beta-prostaglandin F2 in human amniotic fluid and characterization of its production by human gestational tissues. J Clin Endocrinol Metab. 2005;90(7):4244–8. doi: 10.1210/jc.2004-2496 15840748

[85] Lee SE, Romero R, Park I-S, Seong HS, Park C-W, Yoon BH. Amniotic fluid prostaglandin concentrations increase before the onset of spontaneous labor at term. J Matern Fetal Neonatal Med. 2008;21(2):89–94. doi: 10.1080/14767050701830514 18240075

[86] Lee SE, Park I-S, Romero R, Yoon BH. Amniotic fluid prostaglandin F2 increases even in sterile amniotic fluid and is an independent predictor of impending delivery in preterm premature rupture of membranes. J Matern Fetal Neonatal Med. 2009;22(10):880–6. doi: 10.1080/14767050902994648 19544157 PMC4829113

[87] Maddipati KR, Romero R, Chaiworapongsa T, Zhou SL, Xu Z, Tarca AL, et al. Eicosanomic profiling reveals dominance of the epoxygenase pathway in human amniotic fluid at term in spontaneous labor. FASEB J. 2014;28(11):4835–46. doi: 10.1096/fj.14-254383 25059230 PMC4200329

[88] Park JY, Romero R, Lee J, Chaemsaithong P, Chaiyasit N, Yoon BH. An elevated amniotic fluid prostaglandin F2α concentration is associated with intra-amniotic inflammation/infection, and clinical and histologic chorioamnionitis, as well as impending preterm delivery in patients with preterm labor and intact membranes. J Matern Fetal Neonatal Med. 2016;29(16):2563–72. doi: 10.3109/14767058.2015.1094794 26669519 PMC5769707

[89] Rosen EM, van ’t Erve TJ, Boss J, Sathyanarayana S, Barrett ES, Nguyen RHN, et al. Urinary oxidative stress biomarkers and accelerated time to spontaneous delivery. Free Rad Biol Med. 2019;130:419–25. doi: 10.1016/j.freeradbiomed.2018.11.011 30445128 PMC6331226

[90] Eick SM, Ferguson KK, Milne GL, Rios-McConnell R, Velez-Vega C, Rosario Z, et al. Repeated measures of urinary oxidative stress biomarkers and preterm birth in Puerto Rico. Free Radic Biol Med. 2020;146:299–305. doi: 10.1016/j.freeradbiomed.2019.11.003 31704372 PMC6942200

[91] Peiris HN, Romero R, Vaswani K, Gomez-Lopez N, Tarca AL, Gudicha DW, et al. Prostaglandin and prostamide concentrations in amniotic fluid of women with spontaneous labor at term with and without clinical chorioamnionitis. Prostaglandins Leukot Essent Fatty Acids. 2020;163:102195. doi: 10.1016/j.plefa.2020.102195 33137520 PMC8314956

[92] Takahashi N, Okuno T, Fujii H, Makino S, Takahashi M, Ohba M, et al. Up-regulation of cytosolic prostaglandin E synthase in fetal-membrane and amniotic prostaglandin E2 accumulation in labor. PloS one. 2021;16(4):e0250638–e. doi: 10.1371/journal.pone.0250638 33891661 PMC8064594

[93] Durn JH, Marshall KM, Farrar D, O’Donovan P, Scally AJ, Woodward DF, et al. Lipidomic analysis reveals prostanoid profiles in human term pregnant myometrium. Prostaglandins Leukot Essent Fatty Acids. 2010;82(1):21–6. doi: 10.1016/j.plefa.2009.11.002 19954938

[94] Hamberg M, Samuelsson B. On the metabolism of prostaglandins E 1 and E 2 in man. J Biol Chem. 1971;246(22):6713–21. 5126221

[95] Granström E. On the metabolism of prostaglandin F 2 in female subjects. Structures of two metabolites in blood. Eur J Biochem. 1972;27(3):462–9. doi: 10.1111/j.1432-1033.1972.tb01861.x 5050657

[96] Jubiz W, Frailey J. Prostaglandin e generation during storage of plasma samples. Prostaglandins. 1974;7(4):339–44. doi: 10.1016/s0090-6980(74)80089-4 4415345

[97] Smith JB, Ingerman C, Kocsis JJ, Silver MJ. Formation of prostaglandins during the aggregation of human blood platelets. J Clin Invest. 1973;52(4):965–9. doi: 10.1172/JCI107262 4693658 PMC302345

[98] Granström E, Hamberg M, Hansson G, Kindahl H. Chemical instability of 15-keto-13,14-dihydro-PGE2: the reason for low assay reliability. Prostaglandins. 1980;19(6):933–57. doi: 10.1016/0090-6980(80)90127-6 7384561

[99] Bothwell W, Verburg M, Wynalda M, Daniels EG, Fitzpatrick FA. A radioimmunoassay for the unstable pulmonary metabolites of prostaglandin E1 and E2: an indirect index of their in vivo disposition and pharmacokinetics. J Pharmacol Exp Ther. 1982;220(2):229–35. 6948952

[100] Brennand J, Leask R, Kelly R, Greer I, Calder A. The influence of amniotic fluid on prostaglandin synthesis and metabolism in human fetal membranes. Acta Obstet Gynecol Scand. 1998;77(2):142–50. 9512316

[101] Keirse MJNC Turnbull AC. METABOLISM OF PROSTAGLANDINS WITHIN THE PREGNANT UTERUS. Br J Obstet Gynaecol. 1975;82(11):887–93. doi: 10.1111/j.1471-0528.1975.tb00593.x 1191603

[102] Mitchell MD, Keirse MJ, Anderson AB, Turnbull AC. Evidence for a local control of prostaglandins within the pregnant human uterus. Br J Obstet Gynaecol. 1977;84(1):35–8. doi: 10.1111/j.1471-0528.1977.tb12463.x 843469

[103] Romero R, Gonzalez R, Baumann P, Behnke E, Rittenhouse L, Barberio D, et al. Topographic differences in amniotic fluid concentrations of prostanoids in women in spontaneous labor at term. Prostaglandins Leukot Essent Fatty Acids. 1994;50(2):97–104. doi: 10.1016/0952-3278(94)90154-6 8171074

[104] Granström E. On the metabolism of prostaglandin F 2 in female subjects. Structures of two C 14 metabolites. Eur J Biochem. 1972;25(3):581–9. doi: 10.1111/j.1432-1033.1972.tb01731.x 5043324

[105] Ananth CV, Friedman AM, Gyamfi-Bannerman C. Epidemiology of moderate preterm, late preterm and early term delivery. Clin Perinatol. 2013;40(4):601–10. doi: 10.1016/j.clp.2013.07.001 24182950

[106] Hu C, Liu B, Li H, Wu X, Guo T, Luo W, et al. Prostaglandin D2 evokes potent uterine contraction via the F prostanoid receptor in postpartum rats. Eur J Pharmacol. 2018;836:11–7. doi: 10.1016/j.ejphar.2018.08.012 30107163

[107] Clark KE, Austin JE, Stys SJ. Effect of bisenoic prostaglandins on the uterine vasculature of the nonpregnant sheep. Prostaglandins. 1981;22(3):333–48. doi: 10.1016/0090-6980(81)90096-4 7029654

[108] McLaughlin MK, Phernetton TM, Rankin JHG. Prostaglandin D2: Effects on the Rabbit Utero-Placental Circulation. Proc Soc Exp Biol Med. 1979;162(1):187–90. doi: 10.3181/00379727-162-40643 504229

