# Supplementary material for: Prostaglandins in biofluids in pregnancy and labour: A systematic review
Source: PLoS One. 2021 Nov 18;16(11):e0260115. doi: 10.1371/journal.pone.0260115 (PMC8601582; doi:10.1371/journal.pone.0260115)
Supplement: S1 Table — (DOCX) [file pone.0260115.s001.docx]

| # | Searches | Results |
| --- | --- | --- |
| 1 | exp labor/ | 40850 |
| 2 | labour.mp. | 51580 |
| 3 | exp premature labor/ | 47344 |
| 4 | preterm labor.mp. | 8931 |
| 5 | spontaneous labor.mp. | 1922 |
| 6 | exp obstetric delivery/ | 159960 |
| 7 | exp birth/ | 30147 |
| 8 | exp term birth/ | 3627 |
| 9 | exp pregnancy/ | 798469 |
| 10 | (labor adj2 term).mp. [mp=title, abstract, heading word, drug trade name, original title, device manufacturer, device trade name, keyword, floating subheading word, candidate term word] | 2267 |
| 11 | 1 or 2 or 3 or 4 or 5 or 6 or 7 or 8 or 9 or 10 | 953954 |
| 12 | exp prostaglandin/ | 159517 |
| 13 | exp prostacyclin/ | 25635 |
| 14 | prostaglandin metabolite*.mp. | 306 |
| 15 | PGEM.mp. | 1311 |
| 16 | PGFM.mp. | 596 |
| 17 | exp prostanoid/ | 204643 |
| 18 | prostanoids.mp. | 6359 |
| 19 | 12 or 13 or 14 or 15 or 16 or 17 or 18 | 207298 |
| 20 | 11 and 19 | 20718 |
| 21 | exp urine/ | 194295 |
| 22 | exp urine sampling/ | 11727 |
| 23 | exp urinalysis | 108847 |
| 24 | urine samples.mp. | 45512 |
| 25 | 21 or 22 or 23 or 24 | 313085 |
| 26 | 20 and 25 | 201 |
| 27 | exp blood/ | 2652934 |
| 28 | exp serum/ | 290349 |
| 29 | exp plasma/ | 220052 |
| 30 | (blood adj2 maternal).mp. [mp=title, abstract, heading word, drug trade name, original title, device manufacturer, device trade name, keyword, floating subheading word, candidate term word] | 17260 |
| 31 | 27 or 28 or 29 or 30 | 2657286 |
| 32 | 20 and 31 | 2290 |
| 33 | amnio* fluid.mp. | 38408 |
| 34 | 20 and 33 | 906 |
| 35 | limit 34 to human | 641 |
| 36 | limit 32 to human | 1053 |
| 37 | limit 26 to human | 156 |
