# Supplementary material for: Prostaglandins in biofluids in pregnancy and labour: A systematic review
Source: PLoS One. 2021 Nov 18;16(11):e0260115. doi: 10.1371/journal.pone.0260115 (PMC8601582; doi:10.1371/journal.pone.0260115)
Supplement: S2 Table — (DOCX) [file pone.0260115.s002.docx]

| # | Searches | Results |
| --- | --- | --- |
| 1 | exp Labor, Obstetric/ | 45938 |
| 2 | labour.mp. | 31733 |
| 3 | exp Obstetric Labor, Premature/ | 26090 |
| 4 | preterm labor.mp. | 6105 |
| 5 | spontaneous labor.mp. | 1358 |
| 6 | exp Delivery, Obstetric/ | 79518 |
| 7 | exp Parturition/ | 16969 |
| 8 | exp Term Birth | 2936 |
| 9 | exp Pregnancy/ | 888769 |
| 10 | (labor adj2 term).mp. [mp=title, abstract, original title, name of substance word, subject heading word, floating sub-heading word, keyword heading word, organism supplementary concept word, protocol supplementary concept word, rare disease supplementary concept word, unique identifier, synonyms] | 1594 |
| 11 | 1 or 2 or 3 or 4 or 5 or 6 or 7 or 8 or 9 or 10 | 912252 |
| 12 | exp Prostaglandins/ | 100098 |
| 13 | prostacyclin.mp. | 14249 |
| 14 | prostaglandin metabolite*.mp. | 261 |
| 15 | PGEM.mp. | 1051 |
| 16 | PGFM.mp. | 646 |
| 17 | prostanoid.mp. | 5501 |
| 18 | prostanoids.mp. | 5038 |
| 19 | 12 or 13 or 14 or 15 or 16 or 17 or 18 | 108343 |
| 20 | 11 and 19 | 14950 |
| 21 | exp Urine/ | 37160 |
| 22 | urine sampling.mp. | 628 |
| 23 | exp Urinalysis/ | 8048 |
| 24 | urine samples.mp. | 33161 |
| 25 | 21 or 22 or 23 or 24 | 74028 |
| 26 | 20 and 25 | 42 |
| 27 | exp Blood/ | 1095627 |
| 28 | exp Serum/ | 68253 |
| 29 | exp Plasma/ | 26359 |
| 30 | (blood adj2 maternal).mp. [mp=title, abstract, original title, name of substance word, subject heading word, floating sub-heading word, keyword heading word, organism supplementary concept word, protocol supplementary concept word, rare disease supplementary concept word, unique identifier, synonyms] | 8784 |
| 31 | 27 or 28 or 29 or 30 | 1101799 |
| 32 | 20 and 31 | 609 |
| 33 | exp Amniotic Fluid/ | 18680 |
| 34 | 20 and 33 | 437 |
| 35 | limit 34 to humans | 371 |
| 36 | limit 32 to humans | 430 |
| 37 | limit 26 to humans | 35 |
