# Supplementary material for: Prostaglandins in biofluids in pregnancy and labour: A systematic review
Source: PLoS One. 2021 Nov 18;16(11):e0260115. doi: 10.1371/journal.pone.0260115 (PMC8601582; doi:10.1371/journal.pone.0260115)
Supplement: S3 Table — (DOCX) [file pone.0260115.s003.docx]

| **Study** | **Quality Score** |
| --- | --- |
| Karim 1968 | 5 |
| Brummer 1972 | 3 |
| Gutierrez-Cernosek & Levine 1972 | 2 |
| Brummer 1973 | 3 |
| Brummer & Craft 1973 | 2 |
| Hertelendy et al 1973 | 3 |
| Keirse & Turbull 1973 | 6 |
| Salmon & Amy 1973 | 3 |
| Challis et al 1974 | 6 |
| Green et al 1974 | 6 |
| Hamberg 1974 | 1 |
| Hennam et al 1974 | 1 |
| Hibbard et al 1974 | 2 |
| Hillier et al 1974 | 5 |
| Keirse et al 1974 | 3 |
| MacDonald et al 1974 | 4 |
| Singh & Zuspan 1974 | 1 |
| Hillier et al 1975 | 2 |
| Johnson et al 1975 | 6 |
| Pokoly & Jordan 1975 | 6 |
| Dray & Frydman 1976 | 4 |
| Granström & Kindahl 1976 | 5 |
| Keirse et al 1977 | 5 |
| Kinoshita et al 1977 | 6 |
| TambyRaja et al 1977 | 4 |
| Haning et al 1978 | 7 |
| Mitchell et al 1978 | 6 |
| Nieder & Augustin 1978 | 1 |
| Zuckerman et al 1978 | 5 |
| Ghodgaonkar et al 1979 | 4 |
| Mitchell et al 1979 | 4 |
| Satoh et al 1979 | 5 |
| Lewis et al 1980 | 4 |
| Dubin et al 1981 | 9 |
| Sellers et al 1981 | 5 |
| Ylikorkala et al 1981 | 5 |
| Fuchs et al 1982 | 7 |
| Fuchs et al 1982 | 8 |
| Mitchell et al 1982 | 7 |
| Sellers et al 1982 | 7 |
| Sharma et al 1982 | 2 |
| Fuchs et al 1983 | 7 |
| Nieder & Augustin 1983 | 5 |
| Spitz et al 1983 | 5 |
| Husslein & Sinzinger 1984 | 5 |
| Nagata et al 1984 | 7 |
| Reddi et al 1984 | 8 |
| Sellers et al 1984 | 8 |
| Yamaguchi & Mori 1984 | 4 |
| Brennecke et al 1985 | 6 |
| Ogino & Jimbo 1986 | 7 |
| Weitz et al 1986 | 8 |
| Ylikorkala et al 1986 | 6 |
| Berryman et al 1987 | 7 |
| Nagata et al 1987 | 7 |
| Nagata et al 1987 | 6 |
| Romero et al 1987 | 8 |
| Noort et al 1988 | 4 |
| Romero et al 1988 | 8 |
| Sahmay et al 1988 | 7 |
| Noort et al 1989 | 6 |
| Romero et al 1989 | 8 |
| Yamamoto & Kitao 1989 | 8 |
| Mazor et al 1990 | 8 |
| Norman & Reddi 1990 | 5 |
| Fairlie et al 1993 | 8 |
| Hillier et al 1993 | 8 |
| Johnston et al 1993 | 4 |
| MacDonald & Casey 1993 | 8 |
| Romero et al 1993 | 8 |
| Romero et al 1994 | 8 |
| Lindsay et al 1995 | 6 |
| Romero et al 1996 | 7 |
| Ichikawa & Minami 1999 | 7 |
| Mitchell et al 2005 | 6 |
| Lee et al 2008 | 8 |
| Lee et al 2009 | 9 |
| Maddipati et al 2014 | 8 |
| Park et al 2016 | 9 |
| Rosen et al 2016 | 8 |
| Eick et al 2020 | 8 |
| Peiris et al 2020 | 8 |
| Takahashi et al 2021 | 8 |
