# Supplementary material for: Prostaglandins in biofluids in pregnancy and labour: A systematic review
Source: PLoS One. 2021 Nov 18;16(11):e0260115. doi: 10.1371/journal.pone.0260115 (PMC8601582; doi:10.1371/journal.pone.0260115)
Supplement: Attachment — Submitted filename: Response to Reviewers.docx [file pone.0260115.s005.docx]

Dear Dr. Zakar,

Thank you for giving us the opportunity to submit a revised draft of the manuscript “Prostaglandins in biofluids in pregnancy and labour: a systematic review” for publication in *PLOS ONE*. We appreciate the time and effort that you and the reviewers have given to providing your valuable feedback on our manuscript. We have incorporated all the suggestions made by the reviewers. Please see below for a point-by-point response to the reviewer’s comments and suggestions. All page and line numbers refer to the revised manuscript with reviewer comments addressed, and the changes highlighted.

**Journal requirements:**

Comment 1: *Please ensure that your manuscript meets PLOS ONE's style requirements, including those for file naming.*

Response: To ensure that our manuscript meets PLOS ONE’s style requirements the beginning line of each paragraph has been indented, author names on the title page have been reformatted (line 7, page 1), and postal codes and street addresses have been removed from the author affiliations on the title page (lines 9-13, page 1). In addition, the file S1_File has been renamed “PRISMA checklist” in accordance with journal requirement.

Comment 2: *We note that you have included the phrase “date not shown” in your manuscript. Unfortunately, this does not meet our data sharing requirements. PLOS does not permit references to inaccessible data. We require that authors provide all relevant data within the paper, Supporting Information files, or in an acceptable, public repository. Please add a citation to support this phrase or upload the data that corresponds with these findings to a stable repository (such as Figshare or Dryad) and provide and URLs, DOIs, or accession numbers that may be used to access these data. Or, if the data are not a core part of the research being presented in your study, we ask that you remove the phrase that refers to these data.*

Response: We have removed the phrase “not shown” from the manuscript, as it referred to data that is not shown in Figure 2, but which is included in Table 2 and within the manuscript text.

Comment 3: *We note that this manuscript is a systematic review or meta-analysis; our author guidelines therefore require that you use PRISMA guidance to help improve reporting quality of this type of study. Please upload copies of the completed PRISMA checklist as Supporting Information with a file name “PRISMA checklist”*

Response: Our completed PRISMA checklist was previously named S1_File and we have renamed it as “PRISMA checklist” and uploaded it as supporting information.

Comment 4: *Please review your reference list to ensure that it is complete and correct. If you have cited papers that have been retracted, please include the rationale for doing so in the manuscript text, or remove these references and replace them with relevant current references. Any changes to the reference list should be mentioned in the rebuttal letter that accompanies your revised manuscript. If you need to cite a retracted article, indicate the article’s retracted status in the References list and also include a citation and full reference for the retraction notice.*

Response: We have reviewed our reference list and confirmed that we have not referenced any retracted articles. To ensure that the reference list is formatted correctly based on the Vancouver reference style guidelines, we have changed all journal names to their abbreviated versions. Additionally, the reference list was re-ordered slightly to ensure that all references are numbered in the order that they appear in the text (i.e., the reference for Ananth et al. (2013) was moved to position 105 in the reference list (originally position 108) and all the following references moved up one position in the reference list).

**Reviewer’s Comments to the Authors**

**Reviewer 1:**

Comment 1: *At line 286 the authors refer to the short half-life of prostaglandins in blood, which I think is the major factor limiting the clinical application of these studies. This issue is discussed clearly in this section of the paper (lines 281-308) but might be worth mentioning again in the Conclusion.*

Response: We agree with this comment – thank you. Therefore, we have added lines 363-366 (page 29) to the conclusion to reiterate the major limitations identified in this review, including the inability to measure primary prostaglandins in blood due to their short half-lives.

**Reviewer 2:**

Comment 1: *Although the methods of detecting prostaglandins were diverse in different studies, this manuscript will be improved if the authors can summarize the concentrations of prostaglandins measured under the same methods.*

Response: We agree with this suggestion – thank you. Therefore, we have added Table 3 on pages 18-20, which includes the range of prostaglandin concentrations reported under each measurement technique. Lines 156-157 on page 9 refer to this table.

We look forward to hearing from you regarding our submission and to respond to any further questions or comments you may have.

Sincerely,

Eilidh M Wood, Kylie K Hornaday, Donna M Slater
